# The Transition from Animal to Linguistic Communication

**DOI:** 10.1007/s13752-016-0246-2

**Published:** 2016-07-07

**Authors:** Harry Smit

**Affiliations:** Department of Cognitive Neuroscience, Faculty of Psychology and Neuroscience, Maastricht University, Maastricht, The Netherlands

**Keywords:** Animal signals, Babbling, Evolutionary transition, Language evolution, Socially guided learning

## Abstract

Darwin’s theory predicts that linguistic behavior gradually evolved out of animal forms of communication (signaling). However, this prediction is confronted by the conceptual problem that there is an essential difference between signaling and linguistic behavior: using words is a normative practice. It is argued that we can resolve this problem if we (1) note that language evolution is the outcome of an evolutionary transition, and (2) observe that the use of words evolves during ontogenesis out of babbling. It is discussed that language evolved as the result of an expansion of the vocalizing powers of our ancestors. This involved an increase in the volitional control of our speech apparatus (leading to the ability to produce new combinations of vowels and consonants), but also the evolution of socially guided learning. It resulted in unique human abilities, namely doing things with words and later reasoning and giving reasons.

## Introduction

Humans are unique because they are language-using creatures. This observation raises the question of how and why language evolved in only the human species. The precise answer to this question is under discussion, but it is clear that there are many adaptations involved. Walking upright was probably the first step toward linguistic behavior, because it resulted in a change in the position of the larynx (enabling hominids to expand their vocalizing powers) and freed the hands from the constraints imposed by quadrupedal locomotion (they could be used in a wide range of new contexts unrelated to their prior functions, e.g., for gestures such as pointing). The expansion of our vocalizing powers and the use of gestures created opportunities for new ways of communication (e.g., triadic interactions involving pointing facilitated the evolution of linguistic communication about objects). In combination with the capacity to produce consonants and vowels, it resulted in the evolution of the use of words. And the use of words was a first step on the long road to the use of a complex language with a grammar.

Language evolution enabled humans to provide others with information and to seek and acquire information from others. It increased the opportunities for cooperation, but also for cheating, free riding, and so on. In this article I discuss how the transition from animal forms of communication to linguistic behavior was possibly accomplished. How did language evolve out of animal forms of communication? What are possible precursors of linguistic behavior? In order to answer these questions, I first discuss in the following two sections evolutionary explanations of animal communication and elaborate some differences between animal (referential) signals and linguistic behavior. Next I discuss how the use of words may have evolved out of babbling. Babbling occurs at a high frequency in human infancy and is involved in communicative behavior, that is, in turn taking and later triadic interactions involving objects. The vocalizations produced by infants change over time and culminate in the use of words. Yet an explanation of the transition from babbling to using words requires conceptual investigations, since there is an essential difference between babbling and using words. Using words is, and babbling is not, a normative practice, for it involves the correct and incorrect application of rules for the use of words. I shall discuss this conceptual problem and its resolution in the “Using a Language is a Normative Activity” section. In the last three sections before the conclusion I discuss how the transition from babbling to the rule-governed practice of using words may have been accomplished.

## The Evolution of Animal Signals

Evolutionary theorists (e.g., Maynard Smith and Harper 2003; Scott-Phillips 2015) ask how linguistic behavior evolved out of animal forms of communication and use evolutionary theory as an explicatory framework. This framework consists of technical terms made appropriate for studying the fitness effects of communicative behavior, but some of these terms have a non-technical meaning in human practices. This may create confusion if we conflate the rules for the use of technical and ordinary terms. I shall briefly discuss how conceptual investigations clarify the rules for the use of ordinary terms, discuss rules for the use of some ordinary terms involved in human communication, and then continue with technical (evolutionary) definitions.

Conceptual studies investigate the rules for the use of everyday words (for example: “mind,” “state,” “ability,” “body,” “emotion,” “sensation,” “intention,” and “meaning”). These investigations are not empirical but a priori investigations. Consequently, the results may not, at first glance, appear to be novel, for these rules are familiar to a competent user of a language. For example it is not an empirical discovery that emotions, in contrast to abilities (e.g., like the ability to calculate or to understand a language), have genuine duration, intensity, and that some emotions have a characteristic facial expression. Yet although conceptual investigations lead to insights that a competent user of a language can understand, they may result in novel insights if we were misled by a misconception or were not aware of some conceptual features. Suppose we did not realize that, for example, meaning something is not like being in a mental state (like feeling anxious or cheerful). It is then possible that, when we are investigating a segment of our language (the intra-linguistic relationships between meaning, ability, etc.), we will come to know things we had not previously known. We do not learn then new facts about the world, but features of our means of representation. We realize now that when we say that “Meaning is not being in a mental state,” this is not a description of an empirical state of affairs (or a hypothesis), but an expression of an *exclusionary rule* (like “One cannot checkmate in draughts” or “Nothing can be red and green all over at the same time”). This rule or conceptual proposition says that there is no such thing as a mental state of meaning. Notice that the rule is dressed up in the deceptive guise of an empirical description. Also notice that not all conceptual propositions are exclusionary rules. Some are inference rules (e.g., “Red is darker than pink”; if A is red, then the rule licenses us to infer that A is darker than pink); others explanations of meaning in the guise of descriptions of essences, rules for the transformation of descriptions, substitution rules, and so on and so forth.

The terms “signal,” “sign,” and “expression” have both an ordinary meaning and technical meaning (in the context of evolutionary studies). I first discuss some non-technical meanings. Smoke is a sign of fire. We can reliably infer fire from seeing smoke. Humans use the emitted smoke of a fire to signal something, for example danger to another group, or to signal the election of a new Pope. The word “snake” is not a sign of something, but a sign for something. It is not connected to what it represents by causal dependency, but by conventional meaning. There are also examples of non-verbal conventional meaning (Hacker 2013). Nonverbal conventional meaning may be iconic or gestural. Iconic signs may be signs for something (e.g., icons on one’s computer), insignia of something or someone (coats of arms), or signs to *do* something (permitting, forbidding, or requiring one to do something, e.g., stop at the red lights). Gestures, such as nodding or shaking one’s head, or thumbs up or down, likewise signify by convention. There is evidence that, for example, chimpanzees and bonobos also use gestures to signify something (see, e.g., Clay et al. 2015; Genty and Zuberbühler 2015). Expressions of emotions or sensations are not signs of or for something, but *criteria* for sensations or emotions. When we observe, for example, expressions of sensations such as pain, whether these are displayed by animals in their nonverbal behavior or by humans in both nonverbal and linguistic behavior, then we do not infer that an animal has a pain when it expresses pain in its behavior (facial expression, screaming out of pain) or when a human says that he has a pain in his left arm. There is also no external, inductive relation between an emotional expression and what it is an emotion of (by contrast: rain clouds are inductive signs of rain). The nexus between behavior and what it means is non-inductive: the behavior simply manifests what it signifies (while clouds do not manifest rain and smoke does not express fire).

Evolutionary theorists study the problem of how and why animal signals evolved and whether linguistic behavior is rooted in animal signaling. They use a technical definition of a signal. Scott-Phillips (2008) offers the following definition (a slightly modified version from the one used by Maynard Smith and Harper (2003)): a signal is any act or structure that (1) affects the behavior of other organisms; (2) evolved because of those effects; and (3) which is effective because the effect (the response) has evolved to be affected by the act or structure. Two elaborations clarify why this definition is (made) appropriate for evolutionary studies. First, it differentiates between a push and a signal that leads another organism to move, for the signal, in contrast to the push, is selected to affect another organism. Second, the definition also clarifies the role of the receiver in the interaction: the response to the signal has also been selected for. According to the definition, communication occurs when an interaction involving corresponding signal and response is completed. Note that signaling can evolve in both competitive (e.g., fighting over a territory) and cooperative (e.g., cooperative breeding) contexts. Note also that the definition does not mention the concept of “information.”

The definition clarifies how we can distinguish signals from *coercion* and *cues* (see Table 1). Again, coercion and cues are used here as technical terms. An example of a cue is the height of a predator: it is not selected for the purpose of affecting the receiver, but can elicit a response because height may be associated with danger. The push-example mentioned above is an example of coercion but not of a signal, for the receiver is not adapted here to respond to the behavior.

**Table 1:** Distinction between signal, cue, and coercion (adapted from Scott-Phillips 2008)

	Signaler’s behavior evolved for purpose of affecting receiver?	Receiver’s response evolved to be affected by signaler’s behavior?
Signal	Y	Y
Cue	N	Y
Coercion	Y	N

Signals evolved from cues and coercion (Maynard Smith and Harper 2003; Scott-Phillips et al. 2012). They evolved from cues by a process called *ritualization* (Tinbergen 1952). A well-known example is teeth-baring in dogs. According to this hypothesis, teeth-baring evolved into a signal because it could be noticed by the receiver and predicted what the dog was going to do next (namely biting). By noticing the bared teeth, the receiver could anticipate an attack and could, for example, flee or start threatening before being attacked (reducing the fitness costs of injuries). As a result of the process of ritualization (involving selection on both sender and receiver), teeth-baring evolved then into a separate threat signal which is now displayed during contests. In terms of the definition discussed above, teeth-baring was first a cue and later evolved into a signal indicating, at the proximate level, a tendency or fighting ability of an animal. Another example illustrating ritualization is the use of urine to mark territorial boundaries. At first urine was a cue when the focal animal relieved itself because of extreme fear (occurring when the animal left the center of its own territory). The presence of urine could then draw the attention of other animals visiting the area to the presence of the focal individual. As a result of selection on both sender and receiver, this cue could evolve into a signal indicating the territory. In *sensory manipulation* signals evolved from behaviors that were originally coercive (Ryan 1990). Mating displays may have begun by the process of sensory manipulation because of the preference of females for certain objects, enabling males to manipulate them. For example, in many insects males offer females a nuptial gift (e.g., a prey) in exchange for copulation, and this may have begun because females already had a preference for the prey. Another example is the preference of female birds, when they forage, for a certain color (say red) because this color is associated with seeds. Males can then add red to their plumage or build a nest with red objects to exploit this preference and can enhance in this way their reproductive opportunities. Scott-Phillips et al. (2012) argue that there is an important difference between ritualization and sensory manipulation. In ritualization the cue (e.g., bared teeth) manifests a tendency or fighting ability. Receivers attending to the cue have a fitness benefit if they notice the cue. In sensory manipulation, by contrast, attending to the coercive behavior does not need to benefit the receiver because the proto-signal does not manifest a tendency already present in the sender. Because of this difference, the likelihood that signals evolved from cues is greater than that coerced behaviors became signals. There is empirical evidence showing that animal signals evolved more often from ritualization than from sensory manipulation. Is it possible that signals evolved directly, i.e., without first passing through a stage of cueing or coercion? This is unlikely, for direct emergence of communicative signals requires (1) mutations occurring at the same time in both sender and receiver, and (2) that these mutations affect different yet complementary brain structures and processes in the sender and receiver.

Animal signals did not only evolve out of behaviors, but also out of features of the sympathetic and parasympathetic systems because these cause bodily reactions accompanying the expressions of sensations and emotions (e.g., sweating, surface blood-vessel dilatation, thermoregulation). Some bodily reactions obtained a signaling function through the process of ritualization. For example, in chimpanzees “erect hairs” evolved as a signal displayed during contests. In humans “blushing” is a well-known example. It is displayed when someone is embarrassed by his or her misbehavior.

Ethologists have argued in the past that signals evolved to facilitate communication. They thought that ritualized signals (revealing the “tendency” or “motivation” of the sender) were selected because these reduced the costs involved in violent encounters. However, the problem is that ethologists invoked the old notion of group selection (Maynard Smith and Price 1973). They simply assumed that the evolution of ritualized signals was beneficial for the group because it saved lives and prevented injuries (increasing the fitness of the group as a whole). The problems facing the old notion of group selection are well known: groups consisting of individuals sending and responding to “reliable signals” are susceptible to the invasion of a cheater. Suppose that a sender always (whatever his plans) sends the signal that he will attack (or exaggerates a threat signal). Because receivers will always respond to this signal by retreat (in the model of ethologists it is assumed that the signal is always a reliable signal of his tendency to attack), this cheater will invade the population because he has fitness benefits (he wins every contest). This model shows that signals are not simply selected because they benefit the group, as ethologists assumed, but that we have to explain with the aid of evolutionary models, why, on average, reliable signals evolved (see, among others, Johnstone and Grafen 1993; Maynard Smith and Harper 2003). The point to notice is that, because signaling systems are “communicative cooperative,” signaling systems are only stable if signaling benefits both sender and receiver. Otherwise there will be selection in favor of a different response of the individual that does not benefit from the system.

## Meaning Something by a Signal and a Linguistic Utterance

Because evolutionary models discuss the ultimate fitness effects of signals, they do not answer the proximate question whether animal signals have a meaning and convey information. However, it is important to discuss this question, because it clarifies the problem of whether animal signals are possible precursors of linguistic behavior, and what the differences between animal signaling and using words are. I discuss an example that is often used, namely the alarm call of vervet monkeys (see, among others, Seyfarth and Cheney 2003; Wheeler and Fischer 2012; Stegmann 2013).

Senders produce three different calls (to simplify, signaling leopards, eagles, or snakes) and receivers respond to the calls by appropriate hiding behavior. The evolutionary origin of these alarm calls is explicable in terms of inclusive fitness theory.^1^ Suppose that a vervet monkey displays a fear response when it sees or detects a predator. This response, accompanied by a distress call, may then evolve into an alarm call when it results in a hide-response of others (receivers). Kin selection teaches us that an alarm call evolves because it increases inclusive fitness if the receivers are kin. If there is variation in the distress/alarm call, the signal can evolve further into three different calls because the evolving capacity of the sender to produce different calls and of the receiver to discriminate between these calls leads to a further increase in inclusive fitness.

An answer to the question of how we can explain the call at the proximate level is less easy to give since it depends on how we conceive of the meaning of animal signals and linguistic utterances. Two possibilities can be distinguished. According to the first possibility, in both animal and human communication mental acts or processes infuse mere sounds with meaning (although it is assumed that there are essential differences between animal signaling and using a language; see below). Senders of animal signals “mean something” and receivers respond “with understanding” because there are mental acts or processes involved. In the case of the alarm call, it is argued that signals carry “referential information,” for the signals alone (i.e., when receivers do not perceive a predator) elicit the specific response (see discussions in Wheeler and Fisher 2015; Sievers and Gruber 2016). For example, when the sender signals “eagle” receivers look to the sky, showing that they “took something” from the signal. Hence it appears that the signal has a meaning that is understood by the receiver. Three observations are mentioned to substantiate this possibility. First, the production of and response to the call is to a certain extent flexible (hence it may be an example of volitional rather than innate behavior). Moreover the production depends on social context, and the comprehension of the call (more than its production) appears to depend on experience, explaining why older monkeys make fewer mistakes than younger ones (see further Seyfarth et al. 2010). Second, the sender is thought to produce the alarm call in order to inform others that there is an eagle or snake around. It is argued that senders do not simply react to a predator with an alarm call, but respond because they have perceived a snake, for example. And because they have perceived the predator, they know that there is a snake around and can subsequently inform others by the alarm call. In a similar vein, it is argued that receivers, when they hear and respond to the snake alarm call, believe that there is a snake out there or that they expect a snake in their environment when they hear the specific call, since they look for snakes if they hear the snake alarm call. Hence there is evidence showing that receivers can discriminate between things as they believe or expect them to be and things not being so and can exhibit this capacity in their behavior. Third, the alarm call not only informs other individuals that there is a leopard, eagle, or snake nearby, but the vocalizations also inform the hearer that the object is dangerous. This is possible because the call is derived from a fear response: the emotional component of this response enables receivers to make the inference that the object is dangerous.

**Fig. 1:**
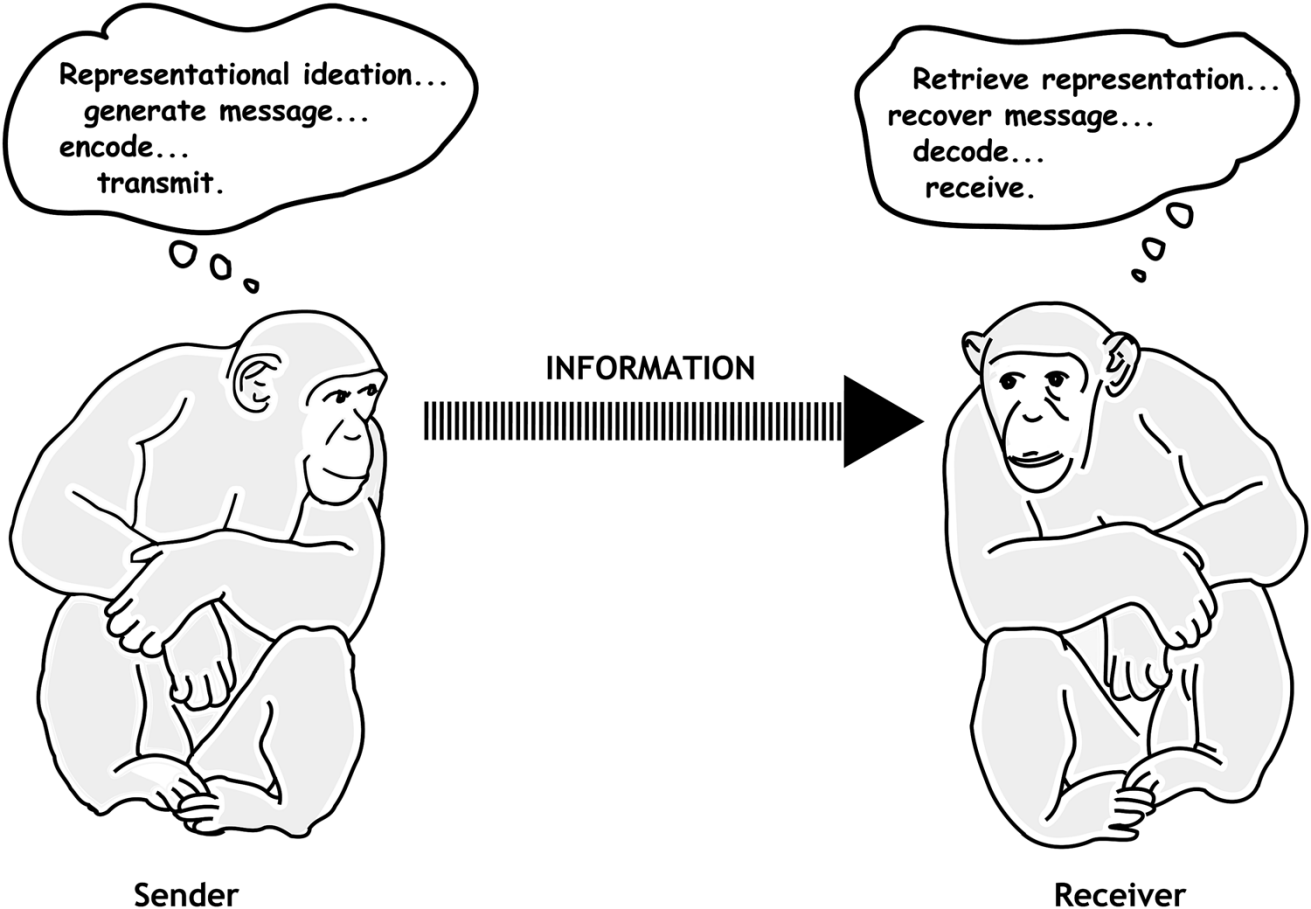
The telementation or code model of animal signaling: a representation by the sender is encoded into a signal that is decoded by the receiver, enabling the receiver to respond (adapted and modified from Rendall et al. 2009)

If “meaning and understanding” are involved in animal signaling, then one can argue that animal signaling may have been a precursor of linguistic behavior. This is further elaborated by arguing that both using a language and signaling involve conveying representations or thoughts by means of signals or linguistic utterances (see Fig. 1). Signals, just like linguistic utterances, evolved according to this possibility as vehicles to communicate representations or thoughts (see the critical discussions in Rendall et al. 2009; Rendall and Owren 2013; Scott-Phillips 2015). According to this so-called telementational or code model communication starts when the sender translates or encodes ideas, representations, or concepts in the medium of sounds for the purpose of communication. These sounds are subsequently translated, interpreted, or decoded by the receiver so that he or she understands what thought or judgment was being communicated by the signal.

If animal signals and linguistic utterances are both vehicles for communicating thoughts, what, then, is the essential difference between animal and linguistic communication? Grice (1989) argued that only humans intentionally signal, i.e., they attempt to induce a change in the mental state of the hearer (this is called the ostensive-inferential model of communication). He argued that, when a speaker means that *p*, he is intending to induce in his addressee the belief that *p* by means of the addressee’s recognition of the intention. When the hearer recognizes the intention, he can infer that *p* was meant. This essential difference between animal and linguistic communication is, according to evolutionary theorists (Scott-Phillips 2015), explicable in terms of the evolution of a metapsychology (involving a theory of mind) only in humans. This metapsychology was, according to them, selected because it enabled humans to cope with the problems of living in large groups. However, whether only humans intentionally signal is currently debated (see Hurford 2007; Wharton 2009; Moore 2016; Scott-Phillips 2016).

According to the second possibility, these models are misguided for conceptual reasons. The reason is that the idea that there are mental acts or processes involved is rooted in the (Cartesian) conception that there are two processes involved in the production of meaningful signals and linguistic utterances, namely mental acts and the physical production of sounds. This so-called dual process theory of meaning is mistaken, for it is not mental acts or processes that give words and sentences meaning, but other linguistic expressions. Words have a meaning because they have been given an arbitrary, conventional purport that can be explained by other expressions, e.g., by paraphrase. And since linguistic utterances, in contrast to animal signals, have a conventional meaning, we can only understand the meaning of words and sentences if we understand the rules for their use. Animal signals do not have a conventional but a natural meaning (or function) that has been selected during the course of evolution.^2^ This difference between natural and conventional meaning is related to the form of signals and linguistic utterances. Alarm calls, in contrast to linguistic utterances, are not arbitrarily structured: they are short with abrupt onsets and broadband noisy spectra. These acoustic features are ideally suited for capturing and affecting the attention and arousal of listeners, resulting in predator-avoidance behavior.

The observation that the meaning of words and sentences is given by other linguistic expressions has an important consequence. It follows that there are no mental acts of meaning that infuse “dead” sounds with life, as is assumed by the code and ostensive-inferential model (see Baker and Hacker 1984; Hacker 2013, Chap. 3),^3^ for there are no separate psychological or mental acts or processes underlying or accompanying speaking, hearing, and understanding. Of course, when we say and mean something, we want our hearer to understand what we mean or meant. What a speaker means by a word or sentence and what a word or sentence means are conceptually linked. But a conceptual link does not imply that there is a separate mental process involved. On the contrary: speakers do not translate their thoughts into a word or sentence (the word or sentence being a vehicle of his or her thought) as is assumed by the code and ostensive-inferential model, for words or sentences are not vehicles for the communication of thought but expressions of thought. Doubts during a conversation about what someone means (“How is that meant?”) are answered by a clarification: we elucidate what we said by paraphrase or by elaborating the implications we had in mind. Hence the doubt is brought about by two or more possible linguistic interpretations, not by concurrent mental processes (in the mind) underlying the utterance of the sentence that was not understood. It is for similar reasons mistaken to assume that the hearer has to infer the meaning, for what is meant is expressed by an utterance he or she already understands. It follows that when children learn the meaning of linguistic utterances, they do not learn to link mental states or processes to words but to couple words to other words and sentences that explain or paraphrase what they say. If they are able to answer the question “How is that meant?” they exhibit an understanding of the relationship between two linguistic expressions, not between dead sounds or signs and mental acts or processes. What they mean is therefore constrained by word meanings, not by mental acts of meaning.

The essential difference between the meaning of animal signals and linguistic utterances is that linguistic utterances have intra-linguistic relationships; meaning and animal signals do not. This enables us to clarify the difference between an alarm call and a linguistic utterance. Suppose that there is evidence that vervet monkeys learn the differences between the three alarm calls and can learn to discern errors when they respond wrongly (hence it is not an innate reaction). We can then still argue that these behavioral data license us to say, when vervet monkeys perceive the snake-alarm call, that they believe or know that there is a snake around. For example if they hide in trees and look to the ground, we can say that they believe that there is a snake around. However, we can now add that animals cannot explain what they mean by the signal since they cannot explain what they are doing (see further Rundle 1997; Hacker 2013). Hence we can distinguish doing something for a reason from behaving because there was a reason. Only humans can act for a reason because they can reason (can explain, give reasons, etc.). A vervet monkey will change the path along which it was sprinting because it noticed a snake there. Yet it did not apprehend the snake then as a reason, for only a creature that acts in the light of reason can act for a reason and can apprehend something as a reason: it is the warrant explaining why it is done. And because such a creature can explain and justify its act by reference to a reason, it can also understand the pros of an action and the cons against it. Hence it can deliberate and can make reasonable choices. Vervet monkeys cannot do that, and when they choose one behavioral option above another as a means to an end, they do not engage in reasoning, for they cannot explain or warrant their behavior by reference to there being a snake on the path. Hence they cannot inform others, after they return to the group, that they changed their path for the reason that they otherwise might have been attacked (they can only signal to others that there is a snake). Creatures can act for a reason if they can reason, i.e., can deduce consequences from assumptions or infer explanations from data (as opposed to only seeing and apprehending something). And this is what human children learn when they learn to reason, i.e., when they learn to answer questions by giving reasons. Vervet monkeys may be prepared for alternative possibilities and can solve problems and discern errors (this does not involve reflectively reconsidering a belief). But they can only solve problems in behaving (they are sensitive to current occurrences in their environment), for solving problems without behaving is restricted to a language-using creature. Vervet monkeys can only recognize, associate, learn, and anticipate, whereas a language-using creature can also reflect, deliberate, reason, infer, ruminate, and so on.

## Using a Language is a Normative Activity

While the question of whether animal signaling is or is not an example of volitional, conative behavior is debated, linguistic behavior is certainly an example of volitional behavior. Moreover, it differs from animal signaling, for only users of a language can explain the meaning of utterances. Children learn to use words correctly. They do not only respond to an explanation of their parents (for example, when their parents point to a fruit in order to explain the word “apple”), but they also learn then what is to be done. Acquiring the ability to use words is acquiring a technique enabling children to participate in a normative practice. A child can be said to have mastered the use of a word (say “red”) if his or her linguistic behavior accords with the acknowledged rules for the correct employment of that word. For example a child understands what red is if he or she points to a ripe tomato for explaining the meaning of “red,” can correct mistakes of others and of him- or her-self, and so on and so forth. In general, whether a child possesses the ability to use words and sentences is determined by testing whether he or she (1) can use a language (e.g., words) correctly, (2) can explain its use correctly, and (3) responds appropriately to its use in context.

These observations raise the problem of how the transition from (natural) signaling to the (conventional) normative practice of using a language was (evolution) and is (ontogenesis) accomplished. The answer I shall discuss below is that language did not evolve directly as an extension of animal signaling (derived from expressions of emotions and sensations), but out of babbling. The idea that babbling is the precursor of linguistic behavior is of course not new. Yet there are three empirical reasons and one conceptual reason why it was and is difficult to see why these vocalizations may have been evolutionary precursors of using words. I briefly discuss the empirical reasons and then discuss the conceptual problem at length.

First, these vocalizations occur at lower amplitudes and are therefore harder to observe than signals such as crying (see, e.g., Oller et al. 2013). Because it seems that these vocalizations are produced accidentally and do not elicit a specific response in receivers, it was thought that they do not have a function. Second, they do not signal need or distress and, hence, appear to be of less importance for infant survival than, for example, distress signals. However, the point to notice is that this does not exclude the possibility that the vocalizations have an inclusive fitness effect in the context of social interactions with the child’s parents (and siblings), e.g., turn taking and later triadic interactions (Smit 2013, 2014). Third, babbling appears to serve only as a preparation for adulthood and does not seem to have an adaptive function in the developmental stage in which it is performed. Although it is possible that babbling does not have (or no longer has) a function during the first year and is only a precursor of later linguistic behavior, we still have to explain how this developmental trajectory was selected during the course of evolution (this problem will not be discussed here).

There is an important conceptual reason why it was difficult to see that babbling may have been an evolutionary precursor of using words. Babbling precedes the use of words, yet there is, as we have seen above, an essential difference between using words and producing these vocalizations. Using words (but not babbling) involves the ability to explain words, and that is a normative activity (we can make and correct mistakes when we use a language). Hence, when a child points to a rose and asks “Red ?” parents can correct him or her by saying “No, pink.” But when an infant utters the babble “Baba,” the parents do not, at first, respond by saying “No, you mean yaya.” I add here the phrase “at first,” for during turn taking and triadic interactions, children are capable of adjusting their vocalizations to what they hear, and parents provide infants with age-appropriate support enabling children to improve their emergent linguistic skills (see the “Learning Words in a Pedagogical Context” section). Hence, for understanding the evolution of the use of words it is essential to explain how during the course of evolution (and during ontogenesis) the transition from babbling to the normative practice of using words was (and is) accomplished. For understanding this transition it is essential to first discuss and resolve three conceptual problems.

First, recall that most animal signals evolved as the outcome of the process of ritualization. This process can be studied with the aid of evolutionary theory because sensations and emotions are characterized by peculiar forms of duration and degree and by their (species-specific) characteristic behavioral expression. Furthermore, there is often a sequence discernable in these expressions, e.g., the opening of the mouth (revealing the teeth) during the expression of aggression in dogs precedes an attack. A dog observing an aggressive opponent can for this reason detect a part (a cue) of the behavioral expression and, hence, can anticipate the attack, resulting in the threat signal “teeth-baring” by the process of ritualization. By contrast, understanding a word, phrase, or sentence, and meaning something by a linguistic expression do not have genuine duration. They are not being in a mental state (it is not like being aggressive for some time) but akin to an ability. If someone is able to V (V represents uses of psychological verbs), her or his behavior that exhibits understanding is constitutive evidence for possession of the ability. The ability consists in being able to explain what words, phrases, and sentences mean. Hence when we listen to someone and understand what she says, then we can explain what she said and respond cogently to her words and can act for the reason that such-and-such was said. That understanding a language is akin to an ability also clarifies why suddenly understanding something is not the beginning of a mental state, but the dawning of a cluster of abilities or possibilities of action and response. We realize then what is meant and what we can do with words. Consequently, language, unlike animal signals, probably did not evolve by the process of ritualization (though its precursors, such as lip smacking and babbling–signaling that an individual is prepared to engage in a social interaction–probably evolved by this process, cf. Van Hooff 1972).

Second, grammar of language is autonomous: explanations of linguistic expressions are given by other linguistic expressions and, hence, remain within language. The meaning of words and sentences are specified by explanations of their meaning. Consequently, how someone has learnt a language is for this reason irrelevant for answering the question whether he can use a language, knows or understands the meaning of words and sentences, and whether he has grasped their use and can explain their meaning. Of course, children learn a language (by imitation, emulation, socially guided learning, etc.; see, e.g., Whiten et al. (2004) for an explanation of these different forms of learning), and saying that grammar is autonomous is not to deny that language learning is an important precondition for understanding a language. But a creature’s pedagogical history is not a criterion for whether a creature has the ability to speak a language. We do not determine whether an alien is speaking a language or just emitting noise by investigating its past—we investigate what it can now do. Whether a child possesses this ability is determined by testing whether he or she can use a language correctly. To have acquired the use of a language, as Wittgenstein (2009 [1953], par. 150) put it, is to have mastered a technique, and the technique mastered is a normative one. Hence, learning a language is important, but whether a child has mastered a language is only tested by the end product.

Third, the observation that training and learning are irrelevant for testing whether someone can use a language correctly requires an explanation of how the transition from babbling to the normative practice of using words was (evolution) and is (ontogenesis) accomplished. I shall argue below that it is essential to conceive of the child as an agent or experimenter. For once the child has passed from the first stages of experimenting with gestures and vocalizations in social and pedagogical contexts to using some words, he or she can ask caregivers what so-and-so is and can ask what such-and-such words mean. These questions are answered by means of explanations of meanings of words. Because these explanations of meaning remain within language, children learn the rules for the use of words and become then participants of a normative practice. Of course, the meaning of some words is explained by pointing to objects (e.g., ostensive definitions), but it is important to recall that explanations by ostensive gestures do not connect language to objects but also remain within language (cf. Hacker 2001, Chap. 9; Baker and Hacker 2005, essay 5; Smit 2014, Chap. 2). These explanations only connect spoken language with the “language of gestures.”

## The Evolution of Consonants and Vowels

Children start to experiment with gestures and vocalizations in the second half of their first year. Well-known transitions occurring during this period are the shifts from babbling to canonical or reduplicated babbling to the use of words. Following Oller (2000), we can distinguish various articulation stages. First, the primitive articulation stage (1–4 months), resulting in the first “goo”- and “coo”-type syllables. At 3–8 months (the expansion/exploratory stage), infants begin to produce marginal syllables, which are slow sequences of consonant–vowel articulation. In the canonical syllable stage (starting at 5–10 months), infants begin to produce fully resonant sounds and faster consonant–vowel alternations, resulting in canonical syllables (e.g., “Bababa”). Children produce the first words at 12–15 months.

An important pattern in babbling is the serial pattern in the case of canonical babbling: the alternation of vowels and consonants. Note that this is also the basic pattern of words. Hence, for understanding the evolution of speech it is essential to answer the question of what led to alternation of vowels and consonants. For if we understand the basic patterns of vowel-consonant alternation, we can explain how the use of more complex patterns was superimposed on this basic pattern resulting in the ability to do various things with words. MacNeilage (1998, 2008) has argued that the basic pattern of speech is the continual rhythmic alternation between an open and closed mouth imposed on the sound production process. The production of the first vowels (e.g., [a]) involves the opening of the mouth, whereas the production of the first consonants (e.g., [b]) involves the closing of the mouth. The assumption here is that during the early stages of language development the infant exerts independent control only of the jaw, while other articulators (lips, tongue, and soft palate) have limited capacity to actively vary their position in the brief span of a syllable. For example, during jaw oscillation, if the tongue is in its resting position, the elevating movements of the mandible will make the lips form a passive constriction and produce a labial consonant, while lowering the jaw produces a central vowel. When the ability to exert control on the articulators increases and infants acquire a greater ability to master fine local movements of articulators (involving the tongue and lips), variegation emerges (involving cortical regulation of speech). Hence, after the stage of reduplicated babbling the original reiterations (“Bababa”) are replaced by utterances in which children produce, for example, different consonants and/or vowels in successive syllables (e.g., from “Mamama” and “Tototo” to “Tomato”).

It is possible that, during our evolutionary history, the production of babbles had a signaling function, for example in the context of mother–child interaction (possibly as an extension of motor patterns involved in sucking, licking, or chewing) or in the context of grooming (it is presupposed here that babbling possibly evolved out of lip smacking). The original small repertoire of signals was later extended as the result of an increase in vocal flexibility into a complex, combinatorial system, enabling children to do various things with words (resulting in linguistic behavior). It is assumed that good imitation skills and later more complex forms of learning were essential here. The crucial step in this scenario is that signals acquired in the end meaning, i.e., the signal “mama” became then the spoken word or sign for “mother” (still later, humans invented a written symbol for “mother”). The signal became a spoken word when humans were able to do something with this utterance, e.g., as a call or exclamation (“Mama!”) resulting in maternal attention. Not surprisingly, there was probably at first no sharp distinction between the first use of words (signs) and signals, because both were produced to draw (or direct) the attention of another (to something).

**Fig. 2:**
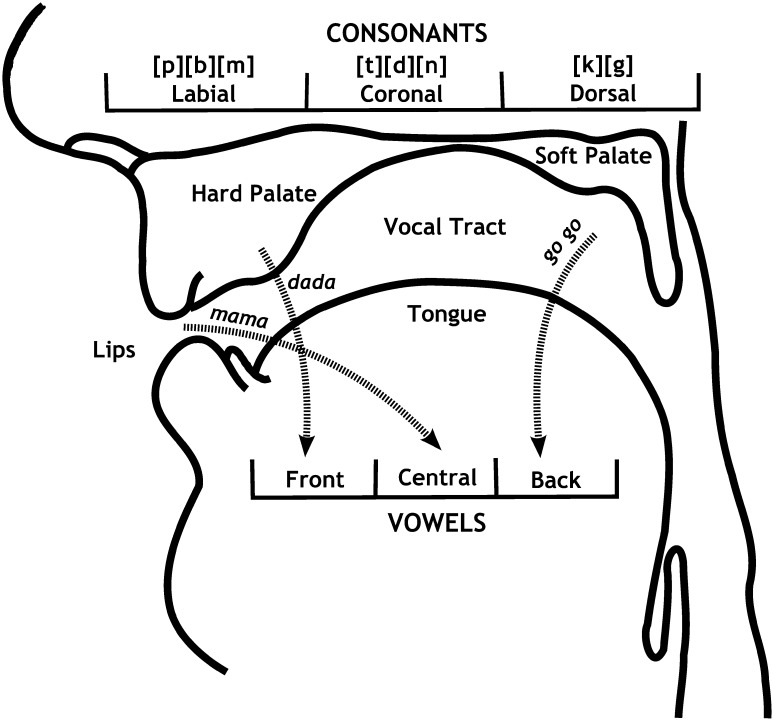
A schematic view of the speech apparatus. The *three arrows* depict three co-occurrence patterns of consonants and vowels, and their connection with articulators such as the lips and the soft palate (adapted and modified from MacNeilage and Davis 2000)

If there were at first morphological and physiological limitations to the production of sounds, then we can expect universal patterns in the production of babbles during the early stages of ontogenesis (and presumably evolution). For example, there is some evidence that there are three intrasyllabic consonant and vowel co-occurrence patterns in babbling (MacNeilage and Davis 2000): (1) labial consonants with central vowels, (2) coronal consonants with front vowels, and (3) dorsal consonants with back vowels (see Fig. 2). Cross-linguistic studies of infant babbling show that these three co-occurrences are present in many cultures. There is also the possibility of a fourth, intersyllabic pattern, namely a tendency to begin a word with a labial stop consonant, and then, after the vowel, to produce a coronal stop consonant. This pattern probably evolved because it is easier to produce a labial consonant than a coronal consonant.

It is unclear, however, whether the rhythmic jaw oscillations, which permit canonical syllables during evolution and ontogenesis were or are shaped by the physiology and neuroanatomy of jaw control. The alternative hypothesis is that these rhythmic movements are the result of a general (whole-organism) developmental pattern, because similar movements occur in the limbs at the same time as the jaw (Iverson et al. 2007; see also Giulivi et al. 2011; MacNeilage and Davis 2011; Oller 2011; Whalen et al. 2011).

Babbling and later linguistic behavior possibly evolved when hominids started to wean children at an earlier time (see Smit 2009, 2013). This became possible when they started to cooperate in the context of hunting large game (but also in the context of digging tubers, etc.). Food was (in the evolving hunter–gatherer societies) then present on a regular basis, enabling hominids to wean children earlier, for humans could use protein-rich meat (but also plant food and tubers) as a supplement to and alternative for maternal milk. Installing earlier weaning, however, required new adaptations. For example “tiny incisors” (milk teeth) evolved enabling children to consume solid food at an early age (milk teeth are absent in other apes). It also required behavioral adaptations enabling mothers and children to adjust their communicative behavior to early weaning. I have suggested that babbling (but also gestures like pointing, etc.) and later linguistic behavior evolved because these behaviors optimized the functioning of food provisioning in the family and group, and, hence, increased the inclusive fitness of individuals living in families and groups. I have also suggested that there may be intragenomic conflicts involved, for there is evidence that mutations of imprinted genes affect the development of babbling and speech.

It is important to notice that I do not, in contrast to others, presuppose that mental acts or processes infuse dead signs with life, for children do not translate thoughts into words and later word combinations, but do things with words (see above). For example, Davis and MacNeilage (2004); see also MacNeilage (2008, Chap. 7), following the misguided ideas of Levelt (1992) and Levelt et al. (1999) and numerous computational psychologists, assume that there are two processes involved in the production of speech. They assume that there is a planning and organization process preceding actual production of speech by the peripheral mechanisms such as the tongue, lips, and jaw. Levelt suggests that (during ontogenesis and evolution) real word production begins when the child starts connecting some particular babble or a modification thereof to some particular lexical concept. At later stages of ontogenesis and evolution these lexical concepts determine the order of words in a sentence. However, the problem is that there are not two processes involved in speaking, for there is no such thing as a mental planning and organization process preceding speech. And one cannot connect a concept to a babble or word; we can give a word a use and we can teach children how to use the words of their language, but children or parents do not thereby connect concepts to words or babbles (when children have mastered the use of a word, we can say that they have acquired the concept expressed by that word). Levelt’s suggestion is akin to supposing that in order for a coin to be worth two euros, we have to connect the value to the coin (see further Bennett and Hacker 2006, 2015).

## Learning Words in a Pedagogical Context

Humans are unique for a biological reason: only in humans an innate ability evolved to learn a language (Kenny 1975, Chap. 1; Kenny 1989, Chap. 10; Smit 2014, Chaps. 6 and 7). But we have additionally seen that learning a language is also a unique human capacity, since the use of language involves the correct and incorrect applications of rules. Learning the use of language was therefore linked to the evolution of asymmetric roles in the family or group: the pupil had to understand that the teacher is the one who is explaining something, whereas the teacher had to check whether the pupil understood what was explained (Csibra and Gergely 2006, 2009). Indeed, empirical studies have shown that socially guided learning is involved in the transition from babbling to doing things with words.

Babbling induces or invites socially guided vocal learning by parents, i.e., when children start to babble, their parents are inclined to join the activity children are at that moment engaged in and respond to their experimental activities and vocalizations with infant-directed speech. Children begin then to respond to the cues and encouragement provided by their parents by improving their skills and vocal capacities.^4^ One study indicates that parents of 11-month-olds produce about 300 infant-directed utterances per hour, and that another 500 utterances per hour, though not directed to the infant, fall on the infant’s ears (see further Locke 2001). That environmental input is important for the development of speech can also be inferred from studies of deaf and hearing-impaired infants. From 6 months onward their babbling is acoustically different from that of hearing infants with a delay in the onset of canonical syllables (see Oller and Eilers 1988; Locke 1993).

Studies have shown that children modify their vocalizations when they are exposed to infant-directed utterances (cf. Locke 1993; Baldwin and Moses 1996; Oller 2000). For example 9-month-old infants can change their babbling to reflect sound patterns in their mother’s speech within minutes. Hence the interactions with caregivers facilitate the development of speech perception and production, presumably because they focus the attention of infants on relevant features of the vocal sounds (and, hence, restrict possibilities). It also enables infants to learn from the consequences of their vocalizing (they can use feedback from the caregiver) and to acquire an understanding of the contingencies of communicative interaction. Interestingly (but obvious to parents and anyone watching parents), there is evidence that parents adjust their utterances to the vocalizations of infants (Oller et al. 2001). Throughout the first year, parent and infant interact and engage in vocal turn-taking. Studies have shown that parents are sensitive to infants’ vocalizations and respond more to speech-like elements in these vocalizations (for example, they are capable of recognizing that their infants’ babbling is more speech-like). They adjust their responses to the different prelinguistic vocalizations in the sense that they increase the complexity of their interactions when infants become more skilled. This is called “acoustic scaffolding,” because parents provide infants with age-appropriate support for their emerging linguistic skills. Hence parents realize that babbling shows that children become experimenters eager to seek information enabling them to improve and refine their vocalizing abilities. For example, 9-month-old infants, interacting with parents producing consonant–vowel (CV) words as a response to their babbling, increased the proportion of syllables with CV structure (Goldstein and Schwade 2008). This was probably not the result of imitating, for the infants did not produce the same phonemes as their mothers used. It may be an example of acquiring the capacity to restructure their own vocalizations into a CV structure matching the ones of their parents. Hence the activity of babbling, together with the responses of the parents, creates socially guided learning.

Infants often babble when they look at and manipulate objects. When parents respond to their babbling, they create opportunities for children to learn links between vocalizations and these objects. Studies show that parents display more verbal responses to object-directed babbling of infants (e.g., by using object labels) resulting in larger vocabularies of the infants at 15 months. Shared attention (both parent and infant attend to an object) and pointing here facilitates learning. Goldstein and Schwade (2009), Goldstein et al. (2010) hypothesize that object-oriented babbling of children signals that they are focusing their attention on the object and are also indicating that they are prepared to learn about the visual features of it. Hence, there is a connection between acoustic learning and learning more about the object to which they are responding vocally, again facilitating the transition from babbling to the use of words.

Word comprehension starts in the second half of the first year. It coincides with object displacement activities (actions in which children move one toy in relation to another, e.g., putting one nesting cup into another, or feeding a doll with a spoon). These object displacement activities are examples of *volitional* behavior, because children orient the object first and then act on it. They can be subdivided into *separations* (disassembling of complex objects into parts) and *constructions* (assembling complex objects out of parts). Feeding a doll with a spoon is also an example of construction, for the child uses here a property of one object in relation to the other. Studies show that separations precede constructions during play, and that the use of words starts when children begin to construct complex objects (see among others Iverson 2010). Note that object construction is less simple than separating complex objects. Also note that, when children only separate objects, it is at first the parent who reconstructs the complex one from the parts so that children can separate them again. The important point to notice is that, as soon as children can construct relations, they can also experiment with varying by creating novel combinations (and by observing the consequences of their actions), enabling them to learn more about the specific properties of objects. Hence in the course of putting objects together, they also start to connect meaning with a referent, i.e., begin to use words. Words and their meanings are learnt in the context of playing with, manipulation, and acting on toys in new and more specific ways, depending in part on developing motor skills. Word comprehension also correlates in this period with the emergence of deitic gestures (e.g., giving, showing, and pointing). The use of these gestures correlates with the first signs of tool use, the categorization of the basis of different features, and imitation of novel acts that were not already in the behavioral repertoire of the child. An important skill children master in the period is joint attention, i.e., they learn to attend to objects and events that adults are watching or indicating; this is seen as a part of the emergence of the system of natural pedagogy (Csibra and Gergely 2006, 2009; see also Tomasello 2008; Smit 2013).

Around 12 months children start to name objects. This is preceded by the brief reproduction of actions associated with objects, e.g., briefly putting a phone to the ear or a cup to the lip. These are called “recognitory gestures” (also called symbolic play schemes), because they seem to know what the object is and what it is used for. The gestures are thought to be a form of nonverbal use of signs because the gestures are brief and stylized in form (e.g., the child who touches cup to lip seems to distinguish between the “recognitory gesture” and “real drinking,” for children are not surprised that there is nothing to drink). Moreover, they sometimes produce the gestures empty-handed. The point to notice is that prior to the emergence of these recognitory gestures, infants act on objects for the purpose of manipulating them. These manipulations are often not specific for the objects. With the appearance of the recognitory gestures, they are able to use action for the purpose of assigning specific meanings to objects. For this reason it is thought that using these gestures also facilitates the acquisition of the ability to convey meaning by words. Parents respond to recognitory gestures by asking questions related to the meaning of the objects. For example, when a child brings a telephone to his or her ear, parents ask: “Are you calling someone?” or “Is that Daddy?” Or if the child touches a cup to the lips of a doll they can ask: “Is she hungry?” Recognitory gestures are transient, for as soon as children acquire the ability to use words, these gestures drop off.

## Doers Become Thinkers

When children acquire the ability to use words they can engage in simple language games. These games are closely linked to what they do and, hence, are context dependent. For instance, they use these words to call their mother (“Mama!”), to name the object they are playing with (“Car”), or to express their wants (“Give!”) or emotions (“Angry!”). These linguistic acts are moves in simple language games.

When their vocabulary expands in their second and third year, they start to make word combinations and later sentences with the aid of a grammar. For example, after they have learnt to replace their natural expressions of the sensation of pain (crying, screaming out of pain) by “Ow,” they learn to extend this expression with “It hurts,” “Have pain,” or “I have pain,” and to apply these predicates to others (“He has pain”). Moreover they learn to indicate where their pain is located (“I have a pain in my toe”) and to explain why they have pain (“I have stubbed my toe”). A similar story can be told in the case of emotions and perceptions. Children learn to extend their natural expressions of, for example, fear (screaming) with linguistic expressions (“I am afraid”) and learn to specify the object of their fear (“A scary dog”). Next children learn that the expressions of emotion may be reasonable or unreasonable. For example, when they are scared because of a dog, their parents may explain to them that the scary dog is a friendly one, and hence, that it is unreasonable to be afraid. By playing as-if games, children learn to pretend, enabling them to understand the difference between being sincere and insincere. They also learn then to deceive others. Their ability to engage in language games involving perception extends when they are taught the use of verbs of perception as operators of names and descriptions of perceptibilia, for example when caregivers ask them questions (“Did you see Daddy?” or “Can you hear the bird?”). They subsequently learn that the objects of perception are sometimes defective (“It looks like …, but …”) because observational conditions are sometimes suboptimal or since the sense organs are (temporarily) malfunctioning. They begin then to understand that the objects of perception and description are sometimes deceptive and look other than they are. Hence the language games involving perception begin with the use of words and, when the perceptual vocabulary expands, end with the use of qualifying observational sentences.

As the result of mastering word combinations and later the use of sentences involving a grammar, children become sensitive to reason. They can explain why they have pain, are angry (“because you took my …”) and correct mistakes (“It appeared red at first, but it is not”). They acquire the ability to reason formally or logically subsequent to having learnt to describe things. For they begin then to understand that sentences are used to describe something that can be true or false. And because descriptions (what are called propositions by logicians) can be true and can be false, they begin to understand the use of logical connectives since this is bound up with the use of “true” and “false” (if, for example, the sentence “Milk is black” is false, milk is not black). Hence, learning the use of true and false is interlaced with learning to use logical connectives. The use of this simple conceptual network (consisting of descriptions, questions, answers, logical connectives, yes or no, true and false) is, in turn, extended with the concept of logical consequences leading to the first forms of formal reasoning and thinking. For the concepts of thinking and reasoning are grammatically interwoven with the use of phrases like “it follows,” “therefore” and “so.” For example, if milk is white, then it follows that it is not black. Importantly, learning all these concepts is embedded in forms of action (see Baker and Hacker 2009, Chap. 7), in what we call saying the same thing, saying something different, denying what we said, contradicting oneself, etc. It is interwoven with justifying what we say and do by reference to reasons and reasoning, but also with understanding, misunderstanding, and not understanding. This conceptual network that children begin to master during their third and fourth year is constitutive of their developing ability to reason and think.

When the ability to reason further expands in their third and fourth year, children can also think conditional thoughts, can think of how things are and how things are not, can conceive of general truths, can think of what does and what does not exist, can use modal expressions and counterfactuals, and so on. Hence, the ability to reason and the use of a tensed language enable children to think of possibilities (to imagine). They start to think about the future and the past; they can think what would happen if…, or what it would be like if … Because they can imagine something different, they begin to understand that one can think falsely. Children can then express thoughts and beliefs and begin to understand that others have thoughts and beliefs. This explains why developmental psychologists have found that children, who can reason and have mastered the use of a tensed language, and so on, can solve false belief tasks at this age, for they can infer what someone believes given what he or she knows in a certain situation.

The ability to reason and to give reasons enables children to pursue goals beyond the immediate environment in space and time, for they can then express thoughts about the future and past. Consequently, there is a difference between the intentional behavior of doers and thinkers, between simple and complex forms of heralding an action. Simple forms are linked to the animal forms of communication, like giving, taking, throwing, wanting, etc. In these cases forming and heralding an action is linked to what children (and animals) will do next (“Give toy” or “Take toy” are followed by the act of giving or taking). Older children (3–4 years old) learn to extend these primitive forms of intentional actions with more complex ones when they have mastered the use of a tensed language, and so on, enabling them to form long-term intentions (e.g., “I will return the toy I borrow from you next Wednesday”). The nexus between expressions of intentions and immediate performance weakens then. Hence, intentional behavior is rooted in goal-directed behavior (also displayed by the other animals), but only children using a tensed language can learn to form and herald long-term intentions. Although there is at first no gap between animals and children (both can pursue goals and can respond to the goal-directed behaviors displayed by others), only children acquire the skill to refer to entities outside the communication situation, to talk about persons who are not present, to focus their attention on something unrelated to current needs and wishes, and so on and so forth.

The shift from doing things with words to reasoning and giving reasons has an important consequence. While we can assume that the first linguistic acts like begging or demanding have been selected because of their inclusive fitness effects, this is less easy to see in the case of the moves in complex language games, like meaning something, intending to do something, reasoning and giving reasons, etc. For when children can mean something, they can explain with the aid of other words what they mean. And when they can do something for a reason, the reason is not a cause of what they intend to do but a warrant (hence they can answer the question why they do something). Thus, the shift that I have discussed from doers to thinkers goes hand in hand with an increase in behavioral flexibility and a decrease of the causal role of genes in children’s behavior. Acquiring an understanding of a complex language frees children from the constraints imposed by genes evolution, for it enables them to pursue self-selected goals.

## Conclusion

I have discussed how linguistic behavior evolved out of babbling. Two innovations were essential. First, linguistic behavior could evolve because the vocalizing powers of our ancestors expanded as the result of enhanced control, mediated by the cortex, of the vocal apparatus. This enabled our ancestors to extend and replace babbling with what became linguistic expressions consisting of different vowels and consonants in successive syllables. Second, speech coevolved with an extension of socially guided learning. Socially guided learning or natural pedagogy probably evolved two to three million years ago when our ancestors started to improve tools and to use them for different purposes. I assume that the use of these tools was, at first, explained by communicative behaviors like ostension, referencing, gaze following, pointing, giving and taking, joint attention and action, and imitation. Notice that these behaviors presuppose a division of labor between tutor and pupil, for teaching and learning are possible if both teacher and pupil understand who is emitting the information and who is supposed to pick up the “intended” information. The child has to observe the eyes of the teacher in order to extract the relevant information, whereas the teacher has to observe the child in order to see whether the information is conveyed. One can imagine that subsequent to the use of the first words, talking to children (infant-directed speech) strengthened the conveying of information. Teaching and learning were a precondition for language evolution, since the rules for the use of words have to be explained to children.

Humans are nowadays unique for a biological reason: only in humans an innate ability evolved to learn the rule-governed use of a language. But learning a language is also a unique human capacity since the use of language is a normative practice. I have argued that the shift from babbling to using words is the result of a developmental and evolutionary transition, for there is an essential difference between babbling and the rule-governed use of words (see also Smit 2014; Smit and Hacker 2014). The essential step in the transition was taken when children could ask questions that were answered by their parents, for they could then learn the meaning of linguistic utterances. They became participants of the normative practice of using a language. Because explanations of meaning remain within language, the pedagogical history (i.e., the gestures and vocalizations that were essential parts of the preceding forms of teaching and learning) dropped off. Of course, it is presupposed here that their parents, as members of a family and group, gave words a use.^5^ Babbling and later using words possibly evolved because, during the early stages of language evolution, these new forms of communicative behavior increased inclusive fitness of individuals living in families and groups. The essential point to notice here is that language evolved because humans could do things with words: to demand, beg, and request; to call people and to respond to calls; to express needs, sensations, and emotions and to respond to the expressions of others; to ask and answer questions; to name things and to describe and to respond to descriptions of how things are; to reason and to give reasons; and so on and so forth. Hence, natural selection could operate on individuals performing simple linguistic acts and later exercising a complex ability. Future studies will reveal the role of (the products of) genes in the transition from babbling to linguistic behavior. They will also clarify the role of genes in the shift from simple linguistic acts (begging, demanding) to the exercise of the ability to use a complex language with a grammar (enabling humans to construct sentences, etc.). Language evolution expanded the horizon of human thought. The horizon of nonhuman animals’ thought is determined by what they can express in their nonverbal behavior, whereas the horizon of human thought is determined by what can be expressed in nonverbal and linguistic behavior. Since language evolved from simple to more complex linguistic behavior, these limits evolved too. What humans could do with words expanded during the course of evolution, resulting in a gap between humans and the other animals.

## Footnotes

1 This requires, of course, far more elaboration than I can give here, because the evolution of alarm calls also depends on the social structure of the group. Vervet monkeys live in multi-male and multi-female groups. Males migrate in this system to another group and then have aggressive encounters with the already present males. The point to notice is that females have more relatives within a group. Given this social structure, female alarm calls (which probably evolved out of an arousal system associated with fear) can increase inclusive fitness by appropriate responses to different predators. Male alarm calls, if present, probably have another origin (see further, e.g., Price et al. 2014).

2 Notice also a similar difference between an animal signal and the smoke signal used by humans (for example, signaling the election of a new Pope). We can only understand the smoke signal if we know the meaning of rules determining what the signal means. Hence the smoke signal is also an example of a conventional signal.

3 Notice that information does not grow or develop in the brain or mind of a child; information has to be acquired by the child (Smit 2014, Chap. 2). Hence, answering the question of whether animal signals transmit information is investigating the problem of whether animals are in possession of information because they perceive and therefore know things, and whether they can transmit this information to others.

4 This requires vocal flexibility. Vocal flexibility, in contrast to flexibility of gesture, was probably limited in the early hominids. It evolved as the result of the selection of variations of genes such as FOXP2 (because the product of this transcription factor is, among other things, involved in the control of fine orofacial movements, enabling hominids to develop articulate speech; see Enard 2011).

5 To give a word a use requires that humans were able to make (vocal) innovations and, hence, requires behavioral and vocal flexibility. Hence we can predict that the genes involved in language evolution (see note 4) increased the vocal powers and enhanced the capacity to learn and memorize words and gestures.
